# Do Chronotypes Influence Problematic Mobile Phone Use and Sleep Quality Among the Doctors? – a Cross-Sectional Study From India

**DOI:** 10.1192/bjo.2022.247

**Published:** 2022-06-20

**Authors:** Prathyusha Gopalakrishnan, Manjula Simiyon, Manikandan Mani, Pradeep Thilakan

**Affiliations:** 1 Pondicherry Institute of Medical Sciences, Pondicherry, India.; 2 Betsi Cadwaladr University Health Board, Wrexham, United Kingdom

## Abstract

**Aims:**

This study aimed to determine the prevalence of problematic mobile phone use and its association with the chronotypes among the doctors of a medical college hospital in Puducherry, India. It also aimed to assess the feasibility of the University of Rochester Modified CAGE Questionnaire as a brief screening tool for problematic mobile phone use.

**Methods:**

This cross-sectional study was conducted at a tertiary care teaching hospital in South India. After obtaining the Institutional ethics committee approval, doctors including consultants, higher trainees, core-trainees, and junior doctors working in various departments were approached and requested to participate in the study. Those who agreed were provided with the participant information sheet and written informed consent was obtained. Part-A of the questionnaire contained requests for personal and professional details and part B had the following questionnaires to assess problematic mobile phone use, chronotypes, phantom ringing and vibration and, sleep quality.

- Problematic use of mobile phone scale (PUMP)
- Reduced Morningness Eveningness Questionnaire (r MEQ)
- Questionnaire for Phantom ringing and Phantom vibration
- The Pittsburgh Sleep Quality Index (PSQI)
- University of Rochester Modified CAGE Questionnaire
- Data were analysed using IBM SPSS Statistics for Windows, Version 20.0 (IBM Corp., Armonk, NY, USA). Nonparametric tests were used as the data were skewed. The data were summarized by frequencies and percentages for categorical variables and median and interquartile range for continuous variables. The chi-square test was used to find the association between two categorical variables. Kruskal Wallis test was used to compare the chronotype with the continuous variables such as CAGE total, PUMP, and PSQ score. Correlation between different continuous variables was studied by using Spearman rank correlations. Kappa statistics were used to evaluate the concordance between PUMP and the University of Rochester Modified CAGE questionnaire.

**Results:**

Neither type (NT) was the most common chronotype (41.5%), followed by morning type (38%) and evening type (20%). Eight (5.6%) doctors had problematic mobile phone use, and 38(26.8%) had poor sleep quality. Evening chronotype (p-value- 0.002), being a female (p-value- 0.014), working in a clinical department (p-value 0.017) and experiencing phantom ringing (p-value- 0.001) had significant association with higher PUMP score. Even though females had a higher median PUMP score, problematic mobile phone use was more among males. University of Rochester Modified CAGE Questionnaire had a sensitivity of 81.73% (73–88.6%), and a specificity of 28.95% (15.4–45.9%).

**Conclusion:**

Doctors should be aware of their mobile phone usage. This study has reiterated the predilection evening chronotype has for behavioral addictions and doctors of evening type should be extra cautious.

